# KASP: a genotyping method to rapid identification of resistance in *Plasmodium falciparum*

**DOI:** 10.1186/s12936-020-03544-7

**Published:** 2021-01-06

**Authors:** Ana Alvarez-Fernandez, María J. Bernal, Isabel Fradejas, Alexandra Martin Ramírez, Noor Azian Md Yusuf, Marta Lanza, Shamilah Hisam, Ana Pérez de Ayala, José M. Rubio

**Affiliations:** 1 Malaria & Parasitic Emerging Diseases Laboratory, National Microbiology Center, Instituto de Salud Carlos III, Madrid, Spain; 2 Department of Clinical Microbiology, Hospital Universitario, 12 de Octubre, Madrid, Spain; 3 Parasitology Unit, Infectious Disease Research Centre, Institute for Medical Research, National Institute of Health, Setia Alam, Selangor, Malaysia

**Keywords:** *Plasmodium falciparum*, KASP, Resistance, SNPs, K13, MDR, Cytb

## Abstract

**Background:**

The emergence and spread of anti-malarial resistance continues to hinder malaria control. *Plasmodium falciparum*, the species that causes most human malaria cases and most deaths, has shown resistance to almost all known anti-malarials. This anti-malarial resistance arises from the development and subsequent expansion of Single Nucleotide Polymorphisms (SNPs) in specific parasite genes. A quick and cheap tool for the detection of drug resistance can be crucial and very useful for use in hospitals and in malaria control programmes. It has been demonstrated in different contexts that genotyping by Kompetitive Allele Specific PCR (KASP), is a simple, fast and economical method that allows a high-precision biallelic characterization of SNPs, hence its possible utility in the study of resistance in *P. falciparum*.

**Methods:**

Three SNPs involved in most cases of resistance to the most widespread anti-malarial treatments have been analysed by PCR plus sequencing and by KASP (C580Y of the *Kelch13* gene, Y86N of the *Pfmdr1* gene and M133I of the *Pfcytb* gene). A total of 113 *P. falciparum* positive samples and 24 negative samples, previously analysed by PCR and sequencing, were selected for this assay. Likewise, the samples were genotyped for the MSP-1 and MSP-2 genes, and the Multiplicity of Infection (MOI) and parasitaemia were measured to observe their possible influence on the KASP method.

**Results:**

The KASP results showed the same expected mutations and wild type genotypes as the reference method, with few exceptions that correlated with very low parasitaemia samples. In addition, two cases of heterozygotes that had not been detected by sequencing were found. No correlation was found between the MOI or parasitaemia and the KASP values of the sample. The reproducibility of the technique shows no oscillations between repetitions in any of the three SNPs analysed.

**Conclusions:**

The KASP assays developed in this study were efficient and versatile for the determination of the *Plasmodium* genotypes related to resistance. The method is simple, fast, reproducible with low cost in personnel, material and equipment and scalable, being able to core KASP arrays, including numerous SNPs, to complete the main pattern of mutations associated to *P. falciparum* resistance.

## Background

Malaria remains an important problem in global public health, being a mayor cause of morbidity and mortality that continues to claim more than 400,000 lives every year [1]. The continuing devastating impact of this disease is partly due to the emergence and spread of resistance to anti-malarials [2].

*Plasmodium* parasites quickly develop resistance to anti-malarials and evade the immune system through mutations in their genome. Since an effective vaccine has not yet been developed, control and surveillance of anti-malarial resistance is crucial for saving lives [3]. *Plasmodium falciparum*, the species that causes most human malaria cases and most deaths, has shown resistance to almost all known anti-malarials [4]. In fact, recently, cases of delayed parasite clearance following treatment with an artemisinin-based combination therapy (ACT) have been reported in the Greater Mekong sub-region. This represent a major threat to the ability to control and treat malaria, since this is the current first line treatment for uncomplicated *P. falciparum* infections [3, 5]. This anti-malarial resistance arises from the development and subsequent expansion of Single Nucleotide Polymorphisms (SNPs) in specific parasite genes [6, 7]. To identify and monitor the propagation of these resistances, SNPs can be detected by molecular analysis. The usual method of resistance detection is the Polymerase Chain Reaction (PCR) and sequencing or digested with restriction enzymes [7]. Moreover, next generation sequencing genotyping is an emerging method of genotyping SNPs that is increasingly being adopted for both diagnosis and research [8].

Despite these advances, rapid, simple, affordable method that can be transferred to the daily clinic are necessary to detect possible resistance in the patient and to a fast population screening of resistance at low cost. It has been demonstrated that genotyping by Kompetitive Allele Specific PCR (KASP), is a simple, fast and economical method that allows a high-precision biallelic characterization of SNPs, as well as insertions and deletions in specific loci, hence its possible utility in the study of resistance in *P. falciparum*.

**Fig. 1:**
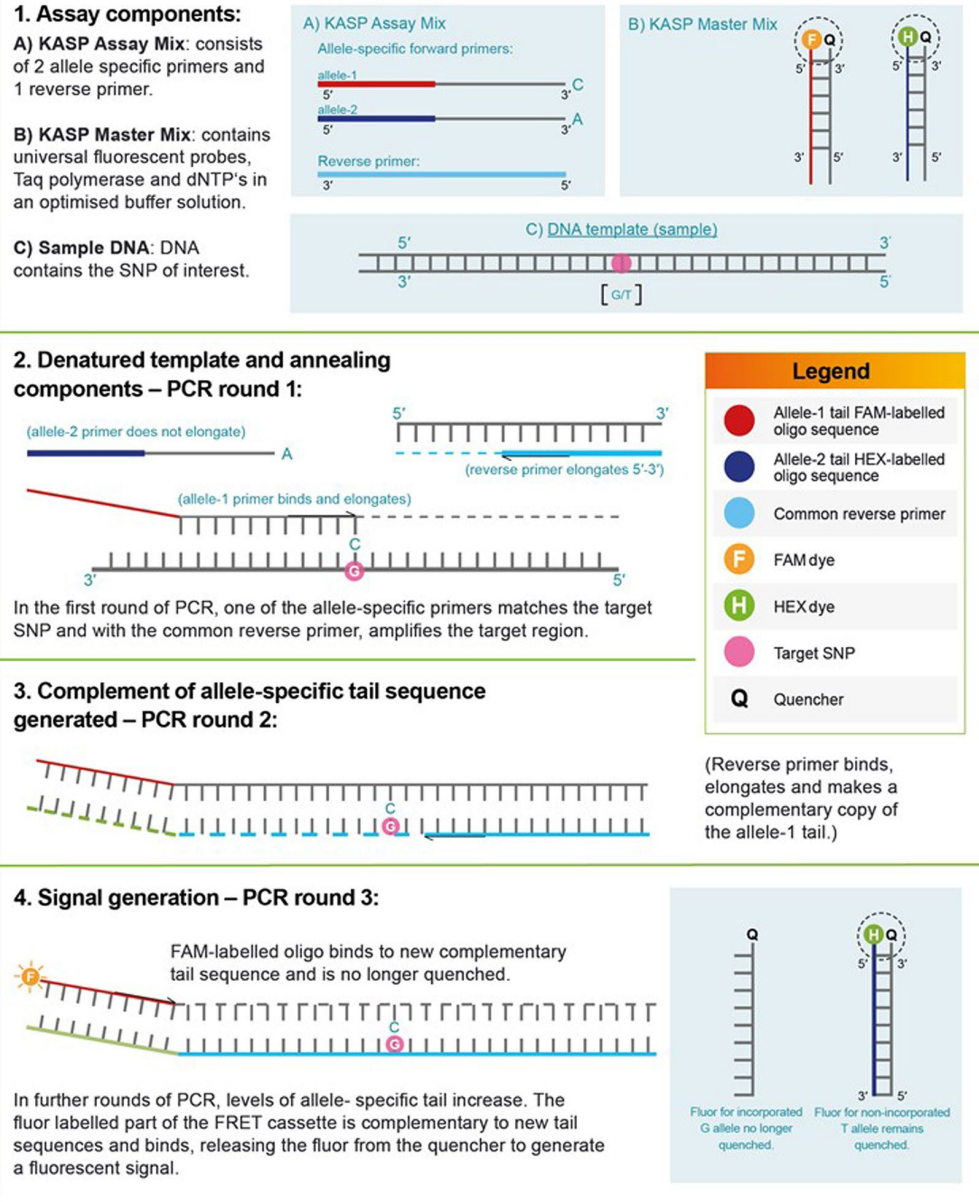
A schematic approach to the KASP mechanism of action

The KASP method introduces fluorescence resonance energy transfer (FRET) for signal generation, where two fluorescent cassettes are used for the identification of allele-specific amplification for a single bi-allelic SNP [9]. The process begins with the first round of the PCR, where one of the allele-specific primers matches the target SNP and, with the common reverse primer, amplifies the target region. The common reverse primer also binds and PCR proceeds, with the allele specific primer becoming incorporated into the template. During this phase, the fluor-labelled oligos remain bound to their quencher-labelled complementary oligos, and no fluorescent signal is generated. As PCR proceeds further, one of the fluor-labelled oligos, corresponding to the amplified allele, is also incorporated into the template, and is hence no longer bound to its quencher-labelled complement. As the fluor is no longer quenched, the appropriate fluorescent signal is generated (Fig. 1) [6].

This method has been used, for example, for the detection of SNPs associated with the efficacy of specific drugs [9]; for genotyping candidate genes associated with the development of genetic diseases, such as Huntington’s disease [10]; or for the genotyping of SNPs associated with G6PD deficiency [11].

Given the importance of rapid response for guidance in the administration of anti-malarial treatment in certain patients, a quick and cheap tool, as KASP, for the detection of drug resistance can be crucial and very helpful for use in hospitals and malaria control programmes. The objective of this study is to verify the possible utility of the KASP technique in the analysis of resistance to anti-malarial drugs. To validate the method, three SNPs involved in most of the resistance of the most widespread treatments have been selected: i) SNP C580Y of the gene that codes for the Kelch13 protein (Gene PF3D7_1343700) that is present in 80% of cases of resistance to artemisinin treatments [12]; ii) SNP Y86N of the *Pfmdr1* gene (gene PF3D7_0523000) that is involved in resistance to different drugs such as quinine, chloroquine, mefloquine, halofantrine, amodiaquine and lumefantrine; the latter frequently used in combination with artemisinin, and where a natural selection of this SNP has been observed after treatment [13–15]; and iii) SNP M133I of the *Pfcytb* gene (gene MAL MYTH 3) that is related to resistance to atovaquone [16] and menoctone [17], being the most frequent mutation in vitro tests.

## Methods

### Sample collection

The *P. falciparum* resistance genotype of those patients who presented a single malaria infection was analyzed as part of a prospective, observational, multicentre study, approved by the Medical and Health Research Ethics Committee (CEIC) of the Hospital Universitario 12 de Octubre (CEIm: 18/021).

The final selection was based on species-specific identification by nested-PCR [18, 19] and only samples with unique infection with *P. falciparum* were chosen for the study.

A total of 112 *P. falciparum* positive samples and 24 negative samples were selected for this assay. In addition, a strain, donated by the Parasitology Department of the Institute for Medical Research in Malaysia with the mutation C580Y of the gene that codes for the Kelch13 protein, was included.

### Sample processing

DNA was extracted from 200 µl of whole blood, collected in ethylenediamine tetraacetic acid (EDTA) tubes, using the QIAamp DNA mini blood kit (QIAGEN^®^, Hilden, Germany) according to the manufacturer’s instructions, resuspended in a final volume of 100 µl of distilled water. The DNA is stored at 4 °C until use, using 5 µl per reaction.

### *Plasmodium falciparum* resistance genotyping

Genotyping of *Pfmdr1*, *Pfcytb* and *PfK13* genes was carried by real time PCR modified from the original methods [20–22]. Reaction mixture consists of 1 × QUANTIMIX HotSplit Probes kit (Biotools, Madrid, Spain) 0.25 μM of each primer (Table 1) and 1 µM EvaGreen^®^ dye (Biotium, Hayward, CA, USA) in a final volume of 20 µl. The thermal cycle was performed in a Qiagen Rotor Gene Q 5 Plex HRM (QIAGEN^®^, Hilden, Germany). The amplification conditions for the *Pfmdr1* and *Pfcytb* genes were an initial cycle of denaturation of 10 min at 95 °C, followed by 45 cycles of 15 s at 95 °C, 15 s at 54 °C and 30 s at 68 °C, ending with a melting step (62 °C to 92 °C). In the case of the *PfK13* gene, the conditions were an initial denaturation cycle of 7 min at 94 °C, followed by 40 cycles of 30 s at 94 °C, 1 min at 60 °C and 1 min at 72 °C, ending with a melting step (62 °C to 92 °C).

**Table 1:** Primers name, gene target, sequence and method for the *P. falciparum* resistance genotyping

Target	Primer name/reference	Method	Seq. 5′→3′
MDR	MDR1-pfmdr1-1FK76T^21^	RT-PCR MDR A and sequencing	GTTGAACAAAAAGAGTACCGCTG
	MDR2-pfmdr1-1RK76T^21^		TCGTACCAATTCCTGAACTCAC
	MDR3-pfmdr1-2FK76T^21^	Sequencing	TTTCCGTTTAAATGTTTACCTGC
	MDR4-pfmdr1-2RK76T^21^		CCATCTTGATAAAAAACACTTCTT
	MDR1-1246-pfmdr1-1FD1246Y^21^	RT-PCR MDR B and sequencing	ATGACAAATTTTCAAGATTA
	MDR2-1246-pfmdr1-1FD1246Y^21^		ACTAACACGTTTAACATCTT
	MDR3-1246-pfmdr1-1FD1246Y^21^	Sequencing	AATGTAAATGAATTTTCAAACC
	MDR4-1246-pfmdr1-1FD1246Y^21^		CATCTTCTCTTCCAAATTTGATA
Cyt B	CytB1-1F^22^	RT-PCR/sequencing	CTCTATTAATTTAGTTAAAGCACAC
	CytB2-1R^22^		ACAGAATAATCTCTAGCACC
	CytB3- 2F^22^	Sequencing	AGCAGTAATTTGGATATGTGGAGG
	CytB4-2R^22^		ATTTTTAATGCTGTATCATACCCT
K13	Pfal-k13-2-2PCR^20^	RT-PCR/sequencing	GCCAAGCTGCCATTCATTTG
	Pfal-k13-3-2PCR^20^		GCCTTGTTGAAAGAAGCAGA

Specificity of amplification was determined by post reaction analysis using the melting temperature (Tm) curve of the amplified fragments (76.8 °C and 76.5 °C for *Pfmdr1* fragment A and B respectively, 79.5 °C for *Pfcytb,* and 82.0 °C for *PfK13*).

Amplicons were purified using Illustra DNA and Gel Band Purification Kit (General Electric Healthcare, England), then sequenced, with its specific primers (Table 1), using Big Dye Terminator v3.1 Kit in an ABI PRISM^®^ DNA Analyzer 3700. All amplified products were sequenced in both directions, twice. Blast tool from NCBI was used to confirm correct target amplification. Multiple nucleotide sequence alignments and analysis were performed using BioEdit version 7.0.5.3 [23] using sequences from 3D7 strain as wild-type for comparison.

### *Plasmodium falciparum* genotyping

*Plasmodium falciparum* genotyping was performed by characterizing two merozoite surface membrane genes (*msp*-*1* and *msp*-*2*) by PCR described elsewhere [24, 25]. Multiplicity of infection (MOI) defined as the number of genetically distinct parasite strains co-infecting a single host, was determined as the largest number of different alleles present at one of the two loci studied.

### Kompetitive allele-specific PCR (KASP)

Sequences flanking SNPs were submitted for KASP ™ assay design to Biosearch Technologies (California, USA). KASP assay was carried out following the instructions of the manufacturer [6]. The reaction mix per reaction consists of 5.1 µl of the KASP Master Mix, which include the two allele specific primers and one reverse primer (Table 2), and 0.138 µl of the Assay Mix, containing universal fluorescent probes, Taq polymerase and dNTPs in an optimized buffer solution.

**Table 2:** Primers and probes used for the SNPs characterization by the KASP method

ID	Primer_AlleleX and Allele Y	Primer_Common
Cyt b M133I	AATTACAGTTGCACCCCAATAACTC GTAATTACAGTTGCACCCCAATAACTT	AACTGCTTTCGTTGGTTATGTCTTACCAT
K13 C580Y	ATACCCCTAGATCATCAGCTATGTG AATACCCCTAGATCATCAGCTATGTA	CTCACCATTAGTTCCACCAATGACATAAA
MDR N86Y	GTGTTTGGTGTAATATTAAAGAACATGA CTGTGTTTGGTGTAATATTAAAGAACATGT	GTACTAAACCTATAGATACTAATGATAATA

To five µl of this reaction mix, five µl of DNA from the sample to be analysed, are added. PCRs and fluorescent readings were performed in a Qiagen Rotor Gene Q 5 Plex HRM (QIAGEN^®^, Hilden, Germany) following the recommended thermal cycling conditions (Table 3).

**Table 3:** Thermal cycle conditions for KASP genotyping reactions

Step	Description	Temperature	Time	Cycles
1	Activation	94 °C	15 min	1 cycle
2	Denaturation	94 °C	20 s	10 cycles
	Annealing/elongation	61–55 °C (drop 0.6 °C per cycle)	60 s	
3	Denaturation	94 °C	20 s	35 cycles
	Annealing/elongation	55 °C	60 s	
4	Read stage (FAM™, HEX™, ROX™)	30 °C	60 s	

### Analysis of KASP genotyping data using cluster plots

To analyse and interpret genotypic data, an Excel sheet is used, although it can also be done using the Thermo Fisher Cloud Genotyping application [26]. In a KASP general assay, a sample that is homozygous for the allele reported by the X-signal oligonucleotide will only generate X-signal fluorescence during the end-point genotyping reaction. This data point will be plotted close to the X-axis, representing a high X-signal and no Y-signal. A sample that is homozygous for the allele reported by the Y-signal oligonucleotide will only generate Y-signal fluorescence during the end-point genotyping reaction. This data point will be plotted close to the Y-axis, representing a high Y-signal and no X-signal. A heterozygous sample will contain both the allele reported by the X-signal oligonucleotide and the allele reported by the Y-signal oligonucleotide. This sample will generate half as much X-signal fluorescence and half as much Y-signal fluorescence as the samples that are homozygous for these alleles. This data point will be plotted in the center of the plot, representing half X-signal and half Y-signal. In falciparum malaria, the situation is more complicated, since, in the blood phase infection, *Plasmodium* is haploid, but *P. falciparum* infections are usually multi-infection so different levels of “heterozygosis” can occur depending on the number of populations present in each infection (MOI). Thus, the points are scattered on the plot but always close to the corresponding axis if only one allele is present, while infections with both alleles will be located in the centre of the plot. Furthermore, to ensure the proper performance of the assay always is useful to include an end-point genotyping reaction without any template DNA as a negative control (referred to as no template control or NTC) that should appear near zero in the cluster plot.

## Results

Analysis for SNPs resistance genotyping showed that only 12 out of 112 samples show any mutations. In all cases this corresponded to amino acid position 86, replacing asparagine with tyrosine (N86Y) of the *Pfmdr1* gene. In the other two genes studied, *Pfcytb* and *PfKelch13*, no mutations were found. The *P. falciparum* strain from Malaysia was found to show the expected C580Y mutation in the *PfKelch13* gene.

The KASP showed that none of the 24 negative samples were amplified, as did NTCs (no template control) included in each reaction. On the contrary, the KASP showed positive results in all the expected samples except one for the SNP of *Pfmdr1*, one more in the case of *Pfcytb* SNP and 3 for *PfK13* SNP. In all cases this lack of results was related to low parasitaemia; in the first two cases, where the samples were not amplified, presented a parasitaemia, quantified by real-time PCR, lower than 0.05 parasites/µl, being less than 1 parasites/µl for the *PfK13*. Despite these results, no statistical correlation has been observed between the level of parasitaemia and KASP values which are between − 0.20 and − 0.67 for wild genotypes and 0.20 for mutated genotypes.

The KASP result for *Pfcytb* M133I was that all of the amplified samples were wild type, as expected. Likewise, in the case of *Pfk13* C580Y, all the samples showed the wild genotype, and only the control strain showed the mutated genotype. In the case of *Pfmdr1* Y86 N, 10 samples gave a mutated genotype and two were characterized with a heterozygous genotype; the rest of the samples showed the wild genotype.

A factor that may also influence is the MOI, which ranges between 1 and 7 in the samples analysed, but no statistical correlation has been observed with the KASP values, obtaining values between − 0.12 and 0.24, except in one case that reaches 0.75.

The reproducibility of the method was analysed by repeating two samples between four and six times in independent tests in the three SNPs, observing that the obtained values did not vary substantially as demonstrated by the low values of the standard deviation (Table 4).

**Table 4:** Reproducibility of the KASP method

Gen	Sample	Type	Arithmetic mean FAM filter	St. Deviation	Arithmetic mean HEX filter	St. Deviation
*Pfmdr1*	222	M	0.08	0.00	0.59	0.04
	223	WT	0.55	0.07	0.14	0.02
*PfK13*	222	WT	0.76	0.04	0.10	0.04
	223	WT	0.76	0.07	0.07	0.008
*Pfcytb*	222	WT	0.92	0.003	0.02	0.001
	223	WT	0.48	0.003	0.08	0.0005

**Fig. 2:**
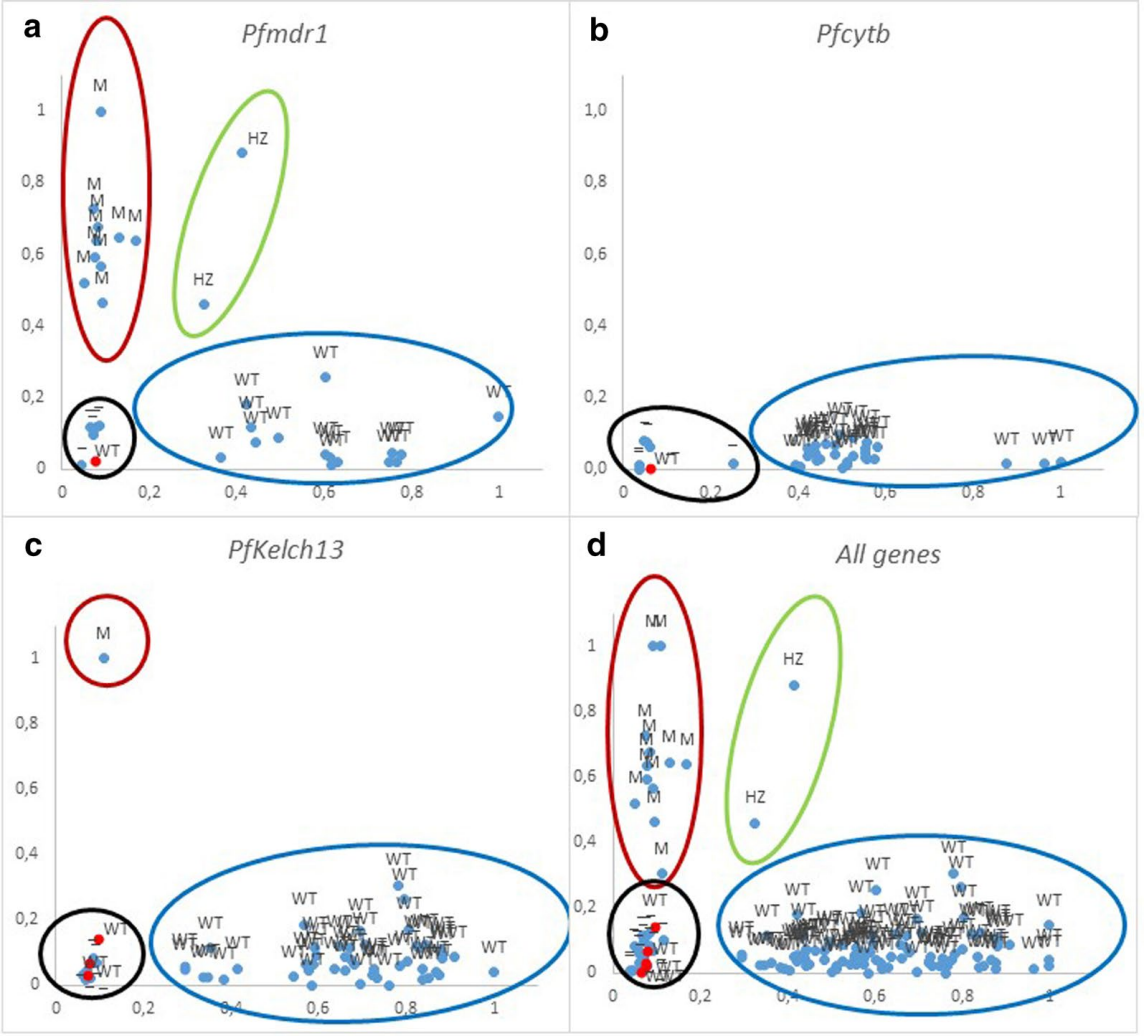
Graphics showing genotypes of the *Pf*mdr1 (**a**), *Pf*cytb (**b**) and *Pf*k13 (**c**) genes, as well as a grouping of the data set (D). Red ovals correspond to WT genotype, blue to mutated genotype, yellow to heterozygous genotypes and green negative controls samples and non-template controls (NTC). Red dots correspond to positives samples that were not detected due to their low parasitaemia. *WT* wild type, *HZ* heterozygous type, *M* mutated type

The expected graphical KASP results should show three clusters, in addition to the negative controls, corresponding to wild type samples, to mutated samples and heterozygous samples (in our case, populations with both alleles). In the three SNPs studied the grouping of the samples according to the expected values are observed. For the Y86 N in the *Pfmdr1* gene, the three clusters are present (Fig. 2a); in the case of the C580Y in the *PfK13*, the cluster of the wild type genotype and that of the mutated strain are observed (Fig. 2c) and in the case of M133I in *Pfcytb* only wild samples are observed (Fig. 2b). By grouping all the SNPs in a single graph, the same result is obtained, with the four clusters well defined (Fig. 2d).

## Discussion

The development and utilization of genetic markers play a pivotal role in the study of malaria pathology in general and in resistance analysis in particular. Among molecular markers, SNPs have become the most promising due to their wide distribution within genomes and suitability for high-throughput automated genotyping [27]. Different methodologies, such as TaqMan, KASP, and rhAmp, have been proposed for the analysis of SNPs using high-resolution automated systems [26]. The Kompetitive Allele Specific PCR genotyping system (KASP™) is a homogeneous, fluorescent, endpoint genotyping technology whose use is expanding in plant genotyping [26, 27]. In malaria, especially in the study of *P. falciparum*, the design of a set of core SNPs array based on this technique could be very interesting for the study of resistance and other SNPs associated with pathology [28]. Firstly, it is necessary to validate this methodology for *Plasmodium* due to the special characteristics of this parasite and the multiple infections it produces.

The system is designed in principle to characterize the presence of bi-alleles in samples of diploid organisms, although its efficacy has also been seen in hexaploid organisms such as wheat [26]. *Plasmodium* in humans is haploid, the only phases where this organism is diploid occurs in the anopheline vector, but the infection, in general, is produced by several genetically distinct parasite strains co-infecting a single host (MOI), therefore, the genotype of a sample for a given gene can cover all allelic variants, being able to present the homozygote of both types or the heterozygote.

The KASP values obtained could be influenced either by the parasitaemia or by the MOI of the samples. Parasitaemia, whose detection limits range from 1 parasites/µl in the case of the analysis of *Pfk13* to 0.05 parasites/µl in *Pfmdr1* and *Pfcytb*, does not influence the KASP values, as demonstrated by the low statistical correlation values (-0.67 to 0.20). Similarly, no correlation was found between the MOI and the KASP values of the sample (-0.12 to 0.75). In both cases, the values obtained are very far from the expected values around ± 1 in the event of a correlation.

In the three SNPs studied, the values obtained correspond to the expected, except in two samples in the Y86N mutation in the *Pfmdr1* gene, which according to KASP we would have both alleles in the sample. This type of case cannot be solved by direct sequencing of the amplified product since in this case only the majority form is detected or in any case, an indeterminacy would occur in the involved nucleotide. The cloning and sequencing of multiple clones or pyrosequencing could resolve this heterozygosis but at a higher cost and with more delay than the KASP. The reproducibility of the technique is very good, showing no oscillations between repetitions in any of the three SNPs analysed (Table 4).

The KASP assays developed in our study were efficient and versatile for the determination of the *Plasmodium* genotypes related to resistance, not being influenced by the parasitaemia of the infection nor by the number of populations involved in it (MOI), and showing high reproducibility.

In conclusion, the method is simple, fast, reproducible and scalable, being able to core KASP arrays developed including numerous SNPs, and, on the other hand, the cost in personnel, material and equipment is lower than with other methodologies.

## Data Availability

All data analyzed during the study are included in this published article. The full datasets of *P. falciparum* genotyping generated during the current study are not publicly available due to its future publication but are available from the corresponding author on reasonable request.

## Abbreviations

Amino acids C: Cysteine
Y: Tyrosine
N: Asparagine
M: Methionine
I: Isoleucine
KASP: Kompetitive allele-specific PCR
MOI: Multiplicity of infection
*Pfmdr1*: *P. falciparum* multi drug resistance gene 1
*Pfcytb*: *P. falciparum* gene cytochrome b gene
*PfK13*: *P. falciparum* Kelch13 gene
SNP: Single nucleotide polymorphism

## References

[1] WHO. World malaria report 2019. Geneva: World Health Organization; 2019.

[2] Otienoburu S, Suay I, Garcia S, Thomas N, Srisutham S, Björkman A (2019). An online mapping database of molecular markers of drug resistance in *Plasmodium falciparum*: the ACT Partner Drug Molecular Surveyor. Malar J..

[3] Cowell A, Winzeler E (2019). The genomic architecture of antimalarial drug resistance. Brief Funct Genomics..

[4] Hartl D (2004). The origin of malaria: mixed messages from genetic diversity. Nat Rev Microbiol.

[5] WHO. Artemisinin resistance and artemisinin-based combination therapy efficacy. Geneva: World Health Organization; 2018.

[6] He C, Holme J, Anthony J (2014). SNP genotyping: the KASP assay. Methods Mol Biol.

[7] Leelawong M, Adams N, Gabella W, Wright D, Haselton F (2019). Detection of single-nucleotide polymorphism markers of antimalarial drug resistance directly from whole blood. J Mol Diagn..

[8] Semagn K, Babu R, Hearne S, Olsen M (2013). Single nucleotide polymorphism genotyping using Kompetitive Allele Specific PCR (KASP): overview of the technology and its application in crop improvement. Mol Breeding.

[9] Suo W, Shi X, Xu S, Li X, Lin Y (2020). Towards low cost, multiplex clinical genotyping: 4-fluorescent Kompetitive Allele-Specific PCR and its application on pharmacogenetics. PLoS ONE.

[10] Ciosi M, Maxwell A, Cumming S, Moss D, Alshammari A, Flower M (2019). A genetic association study of glutamine-encoding DNA sequence structures, somatic CAG expansion, and DNA repair gene variants, with Huntington Disease clinical outcomes. EBioMedicine..

[11] Dombrowski J, Souza R, Curry J, Hinton L, Silva N, Grignard L (2017). G6PD deficiency alleles in a malaria-endemic region in the Western Brazilian Amazon. Malar J..

[12] Zaw M, Lin Z, Emran N (2020). Importance of kelch 13 C580Y mutation in the studies of artemisinin resistance in *Plasmodium falciparum* in Greater Mekong Subregion. J Microbiol Immunol Infect.

[13] Lekana-Douki J, Boutamba S, Zatra R, Edou S, Ekomy H, Bisvigou U (2011). Increased prevalence of the *Plasmodium falciparum* Pfmdr1 86N genotype among field isolates from Franceville, Gabon after replacement of chloroquine by artemether–lumefantrine and artesunate–mefloquine. Infect Genet Evol..

[14] Li J, Chen J, Xie D, Monte-Nguba S, Eyi J, Matesa R (2014). High prevalence of pfmdr1 N86Y and Y184F mutations in *Plasmodium falciparum* isolates from Bioko island. Equatorial Guinea. Pathog Glob Health..

[15] Sisowath C, Strömberg J, Mårtensson A, Msellem M, Obondo C, Björkman A (2005). In Vivo Selection of Plasmodium falciparum pfmdr186N Coding Alleles by Artemether-Lumefantrine (Coartem). J Infect Dis.

[16] Schwöbel B, Alifrangis M, Salanti A, Jelinek T (2003). Different mutation patterns of atovaquone resistance to *Plasmodium falciparum* in vitro and in vivo: rapid detection of codon 268 polymorphisms in the cytochrome b as potential in vivo resistance marker. Malar J..

[17] Blake L, Johnson M, Siegel S, McQueen A, Iyamu I, Shaikh A (2017). Menoctone resistance in malaria parasites is conferred by M133I mutations in Cytochrome b that are transmissible through mosquitoes. Antimicrob Agents Chemother.

[18] Miguel-Oteo M, Jiram A, Ta-Tang T, Lanza M, Hisam S, Rubio J (2017). Nested multiplex PCR for identification and detection of human *Plasmodium* species including *Plasmodium knowlesi*. Asian Pac J Trop Med..

[19] Rubio JM, Post RJ, Docters van Leeuwen WM, Henry MC, Lindergard G, Hommel M. Human malaria diagnosis by seminested mutiplex PCR: Field trials, human blood and dried blood-spot samples. Trans R Soc Trop Med Hyg. 2002;96:199-204. 10.1016/s0035-9203(02)90077-5 12055839

[20] Ariey F, Witkowski B, Amaratunga C, Beghain J, Langlois A, Khim N (2013). A molecular marker of artemisinin-resistant *Plasmodium falciparum* malaria. Nature.

[21] Happi T, Thomas S, Gbotosho G, Falade C, Akinboye D, Gerena L (2003). Point mutations in the *pfcrt* and *pfmdr*-1genes of *Plasmodium falciparum* and clinical response to chloroquine, among malaria patients from Nigeria. Ann Trop Med Parasitol.

[22] Korsinczky M, Chen N, Kotecka B, Saul A, Rieckmann K, Cheng Q (2000). Mutations in *Plasmodium falciparum* cytochrome b that are associated with atovaquone resistance are located at a putative drug-binding site. Antimicrob Agents Chemother.

[23] Hall TA (1999). BioEdit: a user-friendly biological sequence alignment editor and analysis program for Windows 95/98/NT. Nucl Acids Symp Ser..

[24] Robert F, Ntoumi F, Angel G, Candito D, Rogier C, Fandeur T (1996). Extensive genetic diversity of *Plasmodium falciparum* isolates collected from patients with severe malaria in Dakar, Senegal. Trans R Soc Trop Med Hyg.

[25] Snounou G, Zhu X, Siripoon N, Jarra W, Thaithong S, Brown K (1999). Biased distribution of msp1 and msp2 allelic variants in *Plasmodium falciparum* populations in Thailand. Trans R Soc Trop Med Hyg.

[26] Ayalew H, Tsang P, Chu C, Wang J, Liu S, Chen C (2019). Comparison of TaqMan, KASP and rhAmp SNP genotyping platforms in hexaploid wheat. PLoS ONE.

[27] Yang G, Chen S, Chen L, Sun K, Huang C, Zhou D (2019). Development of a core SNP arrays based on the KASP method for molecular breeding of rice. Rice (NY)..

[28] Milner D, Vareta J, Valim C, Montgomery J, Daniels R, Volkman S (2012). Human cerebral malaria and *Plasmodium falciparum* genotypes in Malawi. Malar J..

